# Environmental and microbial factors influencing methane and nitrous oxide fluxes in Mediterranean cork oak woodlands: trees make a difference

**DOI:** 10.3389/fmicb.2015.01104

**Published:** 2015-10-14

**Authors:** Alla Shvaleva, Henri M. P. Siljanen, Alexandra Correia, Filipe Costa e Silva, Richard E. Lamprecht, Raquel Lobo-do-Vale, Catarina Bicho, David Fangueiro, Margaret Anderson, João S. Pereira, Maria M. Chaves, Cristina Cruz, Pertti J. Martikainen

**Affiliations:** ^1^Instituto de Tecnologia Química e Biológica, Universidade Nova de LisboaOeiras, Portugal; ^2^Department of Environmental Science, University of Eastern FinlandKuopio, Finland; ^3^Centro de Estudos Florestais, Instituto Superior de Agronomia, Universidade de LisboaLisboa, Portugal; ^4^Landscape, Environment, Agriculture and Food, Instituto Superior de Agronomia, Universidade de LisboaLisboa, Portugal; ^5^Centre for Ecology and HydrologyPenicuik, UK; ^6^Centre for Ecology Evolution and Environmental Changes, Faculdade de Ciências, Universidade de LisboaLisboa, Portugal

**Keywords:** Mediterranean, oak woodland, methane, nitrous oxide, enzymes, *pmoA*, *nosZ*

## Abstract

Cork oak woodlands (montado) are agroforestry systems distributed all over the Mediterranean basin with a very important social, economic and ecological value. A generalized cork oak decline has been occurring in the last decades jeopardizing its future sustainability. It is unknown how loss of tree cover affects microbial processes that are consuming greenhouse gases in the montado ecosystem. The study was conducted under two different conditions in the natural understory of a cork oak woodland in center Portugal: under tree canopy (UC) and open areas without trees (OA). Fluxes of methane and nitrous oxide were measured with a static chamber technique. In order to quantify methanotrophs and bacteria capable of nitrous oxide consumption, we used quantitative real-time PCR targeting the *pmoA* and *nosZ* genes encoding the subunit of particulate methane mono-oxygenase and catalytic subunit of the nitrous oxide reductase, respectively. A significant seasonal effect was found on CH_4_ and N_2_O fluxes and *pmoA* and *nosZ* gene abundance. Tree cover had no effect on methane fluxes; conversely, whereas the UC plots were net emitters of nitrous oxide, the loss of tree cover resulted in a shift in the emission pattern such that the OA plots were a net sink for nitrous oxide. In a seasonal time scale, the UC had higher gene abundance of Type I methanotrophs. Methane flux correlated negatively with abundance of Type I methanotrophs in the UC plots. Nitrous oxide flux correlated negatively with *nosZ* gene abundance at the OA plots in contrast to that at the UC plots. In the UC soil, soil organic matter had a positive effect on soil extracellular enzyme activities, which correlated positively with the N_2_O flux. Our results demonstrated that tree cover affects soil properties, key enzyme activities and abundance of microorganisms and, consequently net CH_4_ and N_2_O exchange.

## Introduction

Carbon dioxide (CO_2_), methane (CH_4_), and nitrous oxide (N_2_O) are the most important greenhouse gasses (GHG) responsible for global warming. Methane and nitrous oxide contribute 17 and 6% to total global warming (Myhre et al., 2013), respectively. Climate change scenarios for the Iberian Peninsula suggest drier conditions (an average decrease of 20% in precipitation during both winter and summer) and an increase of 40% of the inter-annual variability in the dry period (Meehl and Tebaldi, 2004; Lionello, 2007). This will modify hydrological regimes in Mediterranean-type ecosystems, including the soil’s wet-dry cycles. In the last decades, a decline in cork oak (*Quercus* sp.) has been observed (AFN, 2010) with an increase in tree vulnerability to abiotic and biotic stresses (Garcia-Herrera et al., 2007). Severe and recurrent droughts, as well as intensified wet-dry cycles due to changing climate will alter physical and chemical soil properties, which in turn will affect soil microbiological communities and their activity. Fluctuations of wet-dry cycles have been suggested to have a mechanistic interaction on denitrification through oxygen mediated derepression kinetics, which can contribute to peak N_2_O emissions (Smith and Tiedje, 1979; Betlach and Tiedje, 1981). Moreover, soil moisture can alter the induction time of CH_4_ oxidation in forest soils (Bender and Conrad, 1995). However, relatively little is known about the influence of wet-dry cycles on the fluxes of greenhouse gasses (GHGs) such as CH_4_ and N_2_O in Mediterranean oak forests.

Methane consumption in upland soils is mainly driven by soil methanotrophs, which are unique in their ability to use CH_4_ as carbon and energy sources (Hanson and Hanson, 1996). Methanotrophs are traditionally classified into Type I (aerobic Gammaproteobacteria) and Type II (aerobic Alphaproteobacteria) groups (Hanson and Hanson, 1996). Methanotrophs have the functional gene *pmoA*, which encodes a subunit of particulate methane monooxygenase (pMMO). This gene exists in all methanotrophs with the exceptions of *Methylocella* sp. and *Methyloferula* sp., which have soluble MMO (sMMO; Theisen et al., 2005; Vorobév et al., 2011). Therefore, MMO genes are widely used as a biological marker in molecular ecological studies of methanotrophs (McDonald et al., 2008). Methanotrophs are widely distributed in various environments: such as paddy soils (Bodelier et al., 2000), upland forest soils (Knief et al., 2006; Lau et al., 2007; Mohanty et al., 2007; Kolb, 2009), landfill soils, wetlands (Einola et al., 2007; Siljanen et al., 2011), alpine grassland soils (Abell et al., 2009), and extreme thermoacidophilic environments (Pol et al., 2007; Islam et al., 2008). Soil moisture is important for induction of CH_4_ oxidation and regulation of CH_4_ uptake in soil (Bender and Conrad, 1995; Shrestha et al., 2012). However, methanotrophs are poorly known in temporally dry Mediterranean soils and little is known about how wet-dry cycles influence methanotroph activity and abundance under different vegetation covers (Castaldi and Fierro, 2005; Castaldi et al., 2007; Shvaleva et al., 2014).

The main biological sources of nitrous oxide in soil are nitrification and denitrification processes catalyzed by archaea, bacteria, and fungi (Braker and Conrad, 2011; Thomson et al., 2012; Stieglmaier et al., 2014). Although archaeal nitrifiers and fungal denitrifiers have the ability to produce NO and N_2_O, they lack the capacity for complete N_2_O reduction to N_2_ (Shoun et al., 1992; Kim et al., 2009; Bartossek et al., 2010; Walker et al., 2010; Stieglmaier et al., 2014). Production of N_2_O in forest soils depends on soil characteristics [e.g., moisture, temperature, aeration, pH, soil organic matter (SOM), nitrogen availability] as well as tree species composition (Butterbach-Bahl and Papen, 2002; Skiba et al., 2009; Weslien et al., 2009).

Biological consumption of nitrous oxide in soil is catalyzed by nitrous oxide reductase (NOR) of denitrifying bacteria, which reduces N_2_O to N_2_. Whether soil acts as a sink or a source of nitrous oxide depends on the balance of N_2_O production (nitrification and denitrification) and abundance and activity of denitrifying bacteria carrying NOR. In recent years, the *nosZ* gene, which encodes the catalytic subunit of NOR, has been used as a common molecular marker for analysis of abundance and diversity of denitrifiers capable of N_2_O consumption in soil (Rich et al., 2003; Horn et al., 2006). Novel clade of denitrifiers, recognized as atypical *nosZ* (Sanford et al., 2012) or *nosZ* clade II (*nosZ*-II; Jones et al., 2013), have been recently found to dominate over previously known denitrifiers (Jones et al., 2013). These novel *nosZ*-II carrying denitrifiers have been suggested to contribute significantly to N_2_O consumption/sink activities, since these genes can be correlated with an N_2_O sink (Jones et al., 2014) and a major part of the genomes of these organisms lack genes for N_2_O production (Sanford et al., 2012). However, their respective contribution to the consumption of atmospheric N_2_O is yet to be clearly established.

The heterotrophic soil microbial community is largely responsible for the mineralization of SOM (Bardgett et al., 2002) and availability of carbon and nitrogen regulating microbial processes behind the CH_4_ and N_2_O fluxes. Soil extracellular enzymes play a critical role in SOM decomposition regulating both carbon storage and nutrient supply (Burns and Dick, 2002). Human disturbance and changes in climate can substantially alter the availability of soluble carbon and nitrogen in soil (Nermani et al., 2003). The dry periods represent a significant physiological stress for soil microbial communities (Fierer et al., 2003; Jensen et al., 2003; Gordon et al., 2008; Kardol et al., 2011) and their extracellular enzyme activities (EEAs; Sardans and Penuelas, 2012), which results in reduced SOM turnover and soil nutrient availability (Schmidt et al., 2004; Allison and Treseder, 2008). This can then affect the specific microbial processes driving CH_4_ and N_2_O dynamics.

Previously, we showed that oak trees influence soil properties by increasing the input of litter fall (increase in SOM) which together with changes in soil water content (SWC) can affect net CH_4_ and N_2_O exchange in Mediterranean type ecosystems (Shvaleva et al., 2014). We hypothesize here that trees may affect soil microclimate and prolong influences of wet-dry cycles due to decreased evaporation rates and water uptake from deeper soil layers, which may in turn affect soil extracellular enzymatic activities and therefore have an impact on the functioning of methanotrophs and denitrifying bacteria. The specific hypotheses were: (1) plant cover (cork oak trees) has an effect on abundance of methanotrophs and N_2_O consuming microbes and moreover on N_2_O and CH_4_ fluxes, and (2) in addition to the effect of plant cover, seasonal variation in weather (temperature and precipitation) have an effect on the abundance of methanotrophs and N_2_O consuming bacteria.

## Materials and Methods

### Site Description

The experimental site was located in Herdade da Machoqueira do Grou (39°08′18.29″ N, 8° 19′57.68″ W), 30 km northeast of Coruche, Portugal. The region has a typical Mediterranean climate with hot and dry summers, and moderately cold and mild wet winters. Long-term average meteorological data for this area show that more than 80% of annual precipitation (*ca* 669 mm) occurs between October and May and mean annual temperature is ∼15.9°C (Inst. of Meteorology, Lisbon). The study site is a typical evergreen cork oak open woodland with tree stand age of 50 years and a density of 177 trees h^-1^. The site is certified as *montado and is part of a long-term ecological research project (LTER-Montado)*, which guarantees sustainable management. The natural understory consists of Mediterranean shrub species such as *Cistus salviifolius* L., *Cistus crispus* L., *Lavandula stoechas* L., and *Ulex* spp. and grasses. Two different areas (*ca* 25 m^2^ each) were used to study CH_4_ and N_2_O fluxes, soil properties and abundance of soil microbial communities. These areas were established in the natural understory: under projection of tree crowns (under canopy, hereafter named as UC area); and in large OAs not under projection of tree crowns (hereafter named as OA area). The soil is Cambisol (FAO). The distance between study areas was *ca* 100 m. Standard meteorological data for rainfall (ARG100, Environmental Measurements Ltd., Gateshead, UK), air humidity and temperature (CS215, Campbell, Inc., Logan, UT, USA) were collected over the study period at 30 min intervals and stored using a data logger (CR10X, Campbell Scientific, Inc., Logan, UT, USA).

### Soil Sampling and Temperature

Samples used for determination of seasonal heterogeneity of soil chemical and physical properties and abundance of microbial communities capable of CH_4_ and N_2_O consumption were taken in 2011, May 23rd (end of spring rains), August 31st (dry extreme conditions), October 26th (after the first autumn rain event since August), November 9th (wet extreme) and December 15th (stabilized wet conditions) from triplicated study plots in the UC and OA areas. Soil cores (height 20 cm, diameter 2 cm) were collected from four randomly selected points in the UC and OA areas. For EEA determination, soil samples were additionally taken on July 6th, October 20th, and October 27th in order to increase the power of principal component analysis (PCA). Soil samples were packed in plastic bags and transported to the laboratory in an ice-cooled box. Soil samples for molecular biological analyses were immediately stored at -80°C. Soil temperature at 5 cm depth was measured near to soil gas flux collars by using a digital thermometer. The sample collection was always performed between 09:00 and 13:00 h.

### Soil Chemical Characteristics (C, N, P, SOM, pH, and Electrical Conductivity)

Soil samples for chemical analyses were first sieved (1 mm mesh) and then separated into three parts. One part was used to determine gravimetric SWC (%) by assessing weight loss after drying at 105°C for 24 h. A second part was used to determine nitrate (NO_3_^-^) and ammonium (NH_4_^+^) concentrations by spectrophotometry as described in Fangueiro et al. (2008). The third part of the soil samples was air-dried and analyzed for total soil organic carbon according to Nelson and Sommers (1996) using an Infrared Detection Promacs TOC Analyser (Skalar, Netherlands). SOM content was determined from the soil carbon data using the conventional Van Bemmelen factor of 1.72, i.e., SOM (%) = soil carbon (%) × 1.72 (Nelson and Sommers, 1996). Total nitrogen in the soil was quantified by the Kjeldahl method (Horneck and Miller, 1998), and total phosphorous was determined by the Egner–Rhiem method (Carreira and Lajtha, 1997) using molecular absorption spectrophotometry (Hitachi 2000, Tokyo, Japan). Soil pH was determined in a soil-water suspension (1:10, w/v) with a selective electrode (Micro pH 2001, Criston). Soil electrical conductivity (EC) was measured in a soil-water suspension (1:5, w/v), as described in Fangueiro et al. (2008).

### Enzyme Assays

Soil samples preserved at 4–6°C were used to determine EEA applying photometric and fluorometric micro-plate assays described by Pritsch et al. (2011). Seven EEA were measured, i.e., 1.4-β-xylosidase (Xyl, EC: 3.2.1.37) in presence of MU-xyloside; β-glucuronidase (Glr, EC: 3.2.1.31) in presence of MU-glucoronide; 1,4-β-cellobiosidase (Cel, EC: 3.2.1.91) in presence of MU cellobioydrofuran; *N*-acetyl-β-D-glucosaminidase (Nag, EC: 3.2.1.14) in presence of MU-*N*-acetylglucosamine; β-glycosidase (Gls, 3.2.1.21) in presence of MU-β-glycoside, acid phosphatase (Pho, EC: 3.1.3.2) and laccase (Lac, EC: 1.10.3.2) in presence of 2,2′-azino-bis(3-ethylbenzothiazoline-6-sulphonic acid) ABTS. The main functions of these enzymes are listed in Supplementary Table S2.

### Tree Litter Fall and Root Density

Tree litter fall was determined as described in Shvaleva et al. (2014) with 16 litter baskets placed in two transects across the site with periodic sampling throughout 2011. Root density (dry mass m^-2^) of soil was determined from triplicate soil samples of 0.2 m × 0.2 m × 0.2 m, collected in October 2011. In the laboratory, roots were separated from the soil, washed, and dried at 65°C for 48 h.

### Soil GHG Flux Measurement

Soil-atmosphere net GHG fluxes were measured from six cylindrical collars randomly installed in both UC and OA areas (three replicated study plots per area/treatment). Cylinder collars (polypropylene cylinders, Technical University of Lisbon, Portugal) of 0.3 m diameter were placed at 0.1 m depth into the soil, giving a headspace volume of 0.010 (±0.001) m^3^. The collars were closed with a stainless-steel lid fitted with sample ports (0.006 m diameter), which could be closed and opened by lock valves. The distance between replicates in UC and OA areas was *ca.* 5 m. Flux measurements were done as described in Shvaleva et al. (2014). The chamber was closed at time 0, and samples were taken immediately, at 30 min and after 60 min. Samples of 100 mL were taken from the chambers using a plastic syringe and stored in 20 mL gas vials stopped with butyl rubber septa. Nitrous oxide and CH_4_ concentrations were analyzed at CEH (Edinburgh, UK) by a gas chromatograph (GC, HP5890 Series II, Hewlett Packard, Agilent Technologies UK Ltd., Stockport, UK) fitted with an electron capture detector (ECD) and a flame ionization detector (FID) for N_2_O and CH_4_ analysis, respectively. The flux was calculated based on the slope of a linear regression fitted on data over the measurement time. Calibration of GC was performed with four standard gasses (concentration range: 0.205–1.008 ppm for N_2_O and 1.26–100.9 ppm for CH_4_). GC precision was calculated based on standard gas measurements (*N* = 2–6, depending on number of samples in the GC run). Precision of N_2_O and CH_4_ standards for each four standard gas concentration of all GC runs was ±7 ppb (*N* = 44) for N_2_O and ±70 ppb (*N* = 44) for CH_4_. Minimum detectable fluxes based on precision of GC were 0.94 μg N_2_O-N m^-2^ h^-1^ for N_2_O fluxes and, 11.11 μg CH_4_-C m^-2^ h^-1^ for CH_4_ fluxes with 60 min timescale in chamber volume of 0.010 m^3^ and at 20°C temperature. Discarding these small fluxes (production or consumption) below minimum detectable fluxes would have lead on average to 220 and 53% overestimation of CH_4_ and N_2_O fluxes, respectively. Nitrous oxide and CH_4_ fluxes were compared to each other by calculating CO_2_-equivalent values for both CH_4_ and N_2_O fluxes for making overall comparison of both processes easier. This comparison was made based on radiative forcing of these gasses over 100 years time horizon, factor for CH_4_ was 34 and 298 for N_2_O (Myhre et al., 2013).

### Soil DNA Extraction and Purification

Freeze-dried mortar-homogenized 100 mg soil (stored at -80°C) was used for DNA extraction as described in Siljanen et al. (2011) with slight modification. In brief, after phenol/chloroform/isoamylalcohol extraction, DNA was brownish and therefore it was further purified with PEG6000/NaCl precipitation as previously described by Griffiths et al. (2000). After purification DNA was eluted with 50 μl TE-buffer (Tris-Cl 10 mM, EDTA 1 mM, pH 8.0) and stored at -20°C.

### Quantitative PCR

Presence of PCR inhibiting substances were analyzed by dilution series of extracted DNA with Bacterial 16S rRNA quantitative PCR. It was shown that PCR reaction was not inhibited by undiluted DNA thus samples were used in further analyses. Supplementary Table S3 shows the complete list of primers and conditions used for quantification of microbial communities running CH_4_ and N_2_O consumption. Primer combination A189q (5′-GGNGACTGGGACTTCTGG-3′) and Mb601 (5′- ACRTAGTGGTAACCTTGYAA-3′) targeting *pmoA* gene of Type Ia methanotrophs produced PCR product successfully. For analysis of nitrous oxide consuming bacteria primers targeting *nosZ* genes, nosZ2F (5′-CGCRACGGCAASAAGGTSMSSGT-3′) and nosZ2R (5′-CAKRTGCAKSGCRTGGCAGAA-3′; Henry et al., 2006) primers were used. Both genes were amplified with previously published cycling conditions with Bio-Rad iCycler iQ (Kolb et al., 2003; Henry et al., 2006). Reaction mixtures contained 2x Maxima SYBR Green master mix (Thermo Scientific) and 1 μM of each primer. The quantification of *pmoA* genes was done with cloned fragment of *pmoA* gene according to Siljanen et al. (2011). For quantification of *nosZ* gene genomic DNA of *Pseudomonas aeruginosa* was used. Quantification of both genes was based on a standard curve using 10-fold diluted positive control. Detection limits of qPCR assays were determined from dilution series of positive-control DNA (for *pmoA* 10^8^ to 10^1^ and for *nosZ* 10^6^ to 10^1^) target molecules per reaction. A minimum sensitivity of 10^1^ to 10^2^ target molecules per reaction for each assay was achieved. Amplified PCR products were confirmed by sequencing small clone libraries for both assays.

### Statistical Analyses

A mixed-effect model was used to evaluate the difference of measured variables between UC and OA areas over the timescale studied as previously described in Siljanen et al. (2012). When the data were not normally distributed, they were either square root transformed prior to analysis or non-parametric tests were carried out by performing a comparison on ranks and using Dunn’s test was used for *post hoc* pairwise comparisons. The Pearson Product Moment Correlation coefficient was used to display the strength of the association between pairs of variables. All statistical relationships were considered significant at *P* < 0.05. Statistical analyses were carried out using SigmaStat (SigmaPlot for windows V 11, Dundas Software, Germany), SPSS 17.0 (SPSS, Inc., USA) and R statistical program (R Core Team, 2013).

## Results

### Soil Properties

**FIGURE 1:**
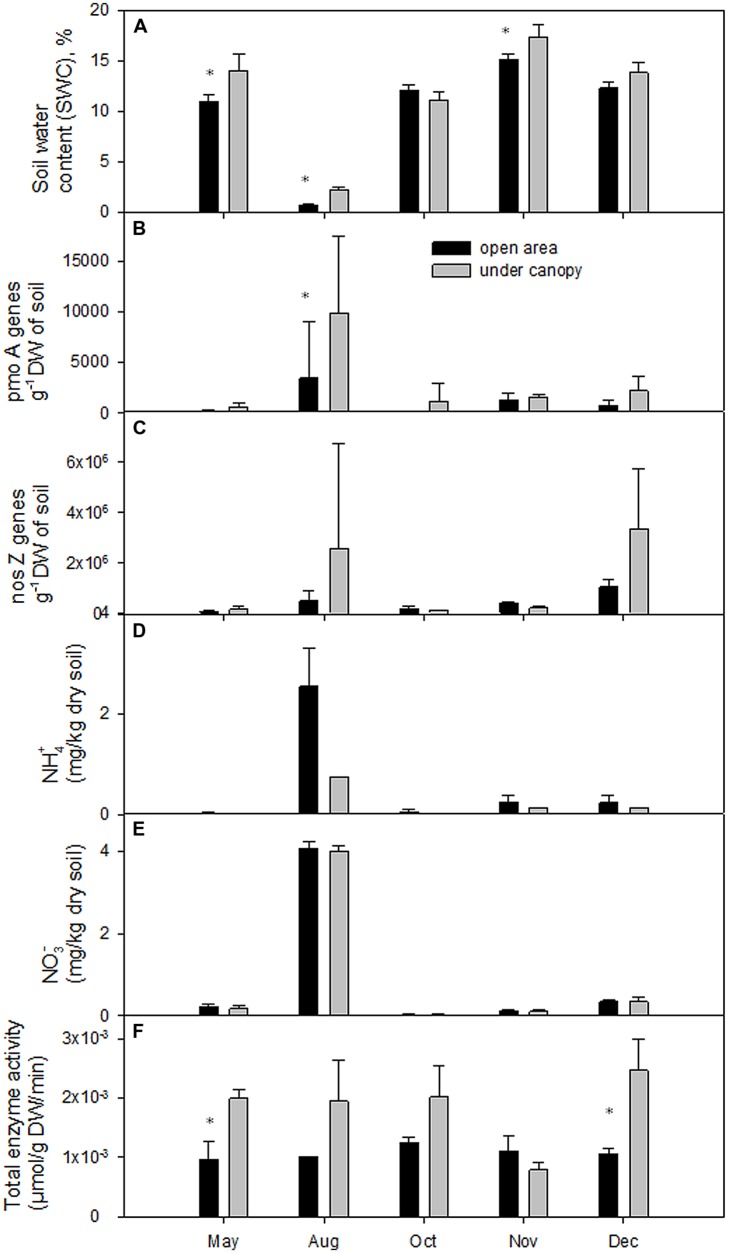
**(A)** Soil water content (SWC,%), **(B)** *pmoA* gene abundance, **(C)** *nosZ* gene abundance, **(D)** soil NH_4_^+^ – N) concentration, **(E)** soil NO_3_^-^ – N concentration, and **(F)** total activities of studied enzymes in the 0–20 cm soil layers of the study areas. Statistical significance (*P* < 0.05) is shown with asterisk.

In 2011 the total annual precipitation was 883 mm and the average air temperature 15.5°C. August was an extremely dry (only 8 mm precipitation) and warm month (Supplementary Table S1). In October, mean air temperature (21°C) was higher than the long-term (1970–2000) average (16°C). Summer conditions extended until mid-October (first rain events occurred on DOY 296 – October 22nd). SWC at 10 cm depth ranged from 2 to 19.5% in the UC and from 0.6 to 16% in the OA (**Figure 1A**). The UC soil was significantly wetter (*P* < 0.001) than the OA soil in May, August, and November (**Table 1**). Soil temperature recorded in the upper 0.05 m varied between 13.7 and 23°C in the UC and between 12.7 and 27.9°C in the OA. The UC had lower soil temperatures than in the OA in May and August, but in December the reverse was true (**Table 1**).

**Table 1:** Statistical significance of the effect of site [under canopy (UC) vs. OA as determined by pair wise comparison] on different soil related parameters: SWC (%), soil temperature, pH, soil organic matter (SOM), soil C/N ratio, carbon (C), nitrogen (N), phosphors (P_2_O_5_), electrical conductivity (EC), NH_4_^+^ – content, NO_3_^-^ – content, ß-glycosidase (Gls), cellobiosidase (Cel), glucuronidase (Glr), glucosaminidase (Nag), phosphatase (Pho), xylosidase (Xyl), total enzymes, *pmoA* gene, *nosZ* gene during the study period.

Parameters significance of effect (site/time)	May	August	October	November	December
SWC ^∗∗∗^/^∗∗∗^	+ ^∗∗∗^	+ ^∗∗∗^	ns	+ ^∗^	ns
Soil temperature ^∗^/^∗∗∗^	-^∗∗∗^	-^∗∗∗^	ns	ns	+ ^∗^
pH	ns	ns	ns	ns	ns
SOM ^∗∗∗^/^∗∗∗^	+ ^∗∗^	+ ^∗∗^	ns	ns	+ ^∗^
C/N	ns	ns	ns	ns	ns
C ^∗^/^∗∗^	+ ^∗∗^	+ ^∗^	ns	ns	+ ^∗^
N ^∗∗^/-	ns	ns	ns	ns	+ ^∗^
P_2_O_5_^∗^/-	+ ^∗^	+ ^∗^	ns	ns	+ ^∗^
EC ^∗^/^∗∗∗^	+ ^∗∗^	+ ^∗∗^	ns	ns	+ ^∗^
NH_4_^+^ -/^∗∗∗^	ns	ns	ns	ns	ns
NO_3_^-^ -/^∗∗∗^	ns	ns	ns	ns	ns
Gls	ns	ns	ns	ns	ns
Cel	+ ^∗^	ns	ns	ns	ns
Glr -/^∗∗^	+ ^∗^	ns	ns	ns	ns
Nag	ns	ns	ns	ns	+ ^∗∗^
Pho ^∗^/^∗^	+ ^∗^	-^∗^	ns	ns	+ ^∗∗^
Xyl -/^∗^	+ ^∗∗^	ns	ns	-^∗∗^	ns
Total enzymes	+ ^∗∗^	ns	ns	ns	+ ^∗∗^
*pmoA* gene -/^∗∗^	ns	+ ^∗^	ns	ns	ns
*nosZ* gene -/^∗^	ns	ns	ns	ns	ns
CH_4_ flux -/^∗^	ns	ns	+ ^∗^	ns	-^∗^
N_2_O flux ^∗^/^∗^	ns	ns	ns	ns	+ ^∗^

*Symbols: ^∗^,^∗∗^,^∗∗∗^ represent statistical significance at *P* < 0.05, *P* < 0.01, and *P* < 0.001, respectively; and ns is not significant at *P* = 0.05, the “tree effect” is shown with +, if under canopy have higher value than open area, and - shows if the under canopy has lower value than the open area. Statistically significant differences of sites and timepoints across all sampling dates are marked with asterisk beside parameter name*.

SOM content in May, August, and December was higher in the UC than in the OA (**Table 1**). The presence of trees in the UC provided twice the input of dry mass m^-2^ (litter fall) compared to the OA (290 g DW m^-2^ y^-1^ vs. 140 g DW m^-2^ y^-1^) and more than twice the root density in the OA (693 ± 70 g DW m^-2^ y^-1^ vs. 314 ± 58 g DW m^-2^ y^-1^). Similarly to SOM, soil electrical conductivity in UC was higher in May, August, and December compared to that in the OA (**Table 1**). No significant differences in soil pH between UC and OA areas were found. Soil total organic carbon and phosphorus (P_2_O_5_) contents were variable and ranged from 1 to 5.8%, and from 4.2 to 30.6 mg kg^-1^ DW, respectively; these contents in the UC area were significantly higher in May, August, and December (**Table 1**). No differences in total soil N, and content of NO_3_^-^ – N and NH_4_^+^ – N content between the UC and OA were observed.

### Quantification of *pmoA* and *nosZ* Genes

The methanotrophic *pmoA* gene abundance was detected throughout the study period in the UC and OA areas and ranged from 3 × 10^2^ to 16 × 10^3^ *pmoA* genes g^-1^ DW and from 8 × 10^1^ *pmoA* genes g^-1^ DW to 10 × 10^3^ *pmoA* genes g^-1^ DW, respectively. In the UC site Type Ia *pmoA* gene copy numbers were more than 10 times higher in August compared to other periods of study (**Figure 1B**). Under the extreme dry conditions encountered in August, the abundance of methanotrophs in the UC was significantly higher than in OA (*P* < 0.05, **Table 1**). Our data showed positive correlations between *pmoA* gene abundance and soil NH_4_^+^ content in OA (Pearson’s *r* = 0.521, *P* < 0.05) and in UC with NO_3_^-^ (*r* = 0.65, *P* < 0.01) content, i.e., the number of methanotrophs increased with increasing mineral nitrogen content (**Figures 1D,E**). Moreover, a negative correlation was observed between *pmoA* gene abundance and CH_4_ flux (*r* = -0.54, *P* < 0.05) in the UC and with total nitrogen (*r* = -0.52, *P* < 0.05) in the OA.

Quantitative PCR with primers q189A/Mb601 targeting Type Ia methanotrophs was the only assay producing PCR products successfully. Other phylogenetic methanotroph groups (MOB amplified in nested PCR with A189/A682/mb661 primers and quantitative PCR with USCα, Type Ib, Type II and *Methylocella* sp. primers) showed only negligible PCR products.

The *nosZ* gene abundance in the UC and OA varied in range from 6 × 10^4^ to 7.3 × 10^6^ *nosZ* genes g^-1^ DW and from 1 × 10^5^ to 1.3 × 10^6^ *nosZ* genes g^-1^ DW, respectively (**Figure 1C**). Under summer drought (August) and stabilized wet conditions in winter (December, SWC around 15%) the number of *nosZ* gene abundance increased in the UC more than 18 times compared to other seasons. However, no differences in the *nosZ* gene abundance between the UC and OA were observed during the study. A negative correlation between *nosZ* gene abundance, and N_2_O flux (*r* = -0.59, *P* < 0.05) was observed in the OA site, but not in the UC site.

### Soil Enzyme Activities

Total enzyme activities were significantly higher in the UC area in May and December (**Figure 1F**). Enzyme activities did not correlate with gene copy numbers or CH_4_ fluxes, but correlated with N_2_O fluxes. In the UC area, N_2_O flux correlated positively with total enzyme activity (*r* = 0.60, *P* < 0.05), with Glucuronidase activity (*r* = 0.58, *P* < 0.05), with Glucosaminidase activity (*r* = 0.67, *P* < 0.01), and with phosphatase activity (*r* = 0.56, *P* < 0.05), whereas in OA site, N_2_O fluxes had a positive correlation with phosphatase activity (*r* = 0.58, *P* < 0.05).

### Soil Net CH_4_ and N_2_O Fluxes

Results showed that the soil acted mainly as a net sink for CH_4_, however there were also periods of net CH_4_ emissions. During the study period CH_4_ fluxes ranged from -12.3 to 8.6 μg Cm^-2^ h^-1^. Methane emissions were observed in May in both UC and OA, and in August in the OA only (**Table 2**). The difference in CH_4_ flux between areas was highest in October, when the CH_4_ uptake in the OA was higher than in the UC, and in December, when on the contrary, CH_4_ uptake in the OA was lower than in the UC. However, the tree cover had not a general effect on CH_4_ flux when all time-points were included to the analysis (Mixed-effect model: d.f._1_ = 1, d.f._2_ = 20, *P* = 0.655). Methane fluxes correlated positively with soil temperature both in the OA (*r* = 0.75, *P* < 0.01), and UC areas (*r* = 0.79, *P* < 0.001). Methane fluxes also correlated positively with organic matter (*r* = 0.55, *P* < 0.05), CN-ratio (*r* = 0.57, *P* < 0.05) and total carbon (*r* = 0.67, *P* < 0.01) in the UC area. Mean CH_4_ fluxes, shown as CO_2_-equivalent fluxes were not different between areas (**Table 2**).

**Table 2:** Soil CH_4_ (μg CH_4_-C m^-2^h^-1^) and N_2_O (μg N_2_O-N m^-2^h^-1^) fluxes measured at the study site from May to December, 2011.

Fluxes	Site	May	August	October	November	December	Mean of fluxes (CO_2_^eq^)
CH_4_ [μg CH_4_-C m^-2^h^-1^]	OA	2.56 ± 4.4	1.09 ± 0.43	-12.75 ± 0.99 ^a^	-4.71 ± 0.32	-6.68 ± 0.89 ^a^	-4.10 ± 0.60 (-217 ± 32)
	UC	2.22 ± 3.39	-1.1 ± 0.57	-8.29 ± 3.3 ^b^	-5.69 ± 0.57	-11.09 ± 0.73 ^b^	-4.78 ± 1.21 (-186 ± 16)
N_2_O [μg N_2_O-N m^-2^h^-1^]	OA	-0.77 ± 0.04	0.22 ± 0.48	0.78 ± 2.08	-2.41 ± 0.63	-5.5 ± 0.93 ^a^	-1.54 ± 0.76 ^a^ (-720 ± 204)
	UC	-0.55 ± 0.34	1.35 ± 0.99	-0.66 ± 1.32	-1.4 ± 0.94	4.42 ± 1.12 ^b^	0.59 ± 0.64 ^b^ (276 ± 174)

*Carbon dioxide equivalents (CO_*2*_^eq^, μg CO_*2*_^eq^ m^-2^h^-1^) were calculated by multiplying the flux with Global Warming Potential in time-horizon of 100 years (CH_*4*_ = 34; N_*2*_O = 298, Myhre et al., 2013). Values are mean ± SE (*n* = 3). December, 2011. Statistical significant differences (*P* < 0.05) between OA and UC area is shown with different letters*.

There was both net uptake and net release of N_2_O occurring and the flux varied from -6.5 to 6 μg N_2_O-N m^-2^ h^-1^ (**Table 2**). The most pronounced difference between areas was observed in December when the UC had N_2_O release but the OA showed N_2_O uptake. The tree cover had a general effect on CH_4_ flux when all time-points were included in the analysis (**Table 2**; Mixed-effect model: d.f._1_ = 1, d.f._2_ = 20, *P* < 0.05). Nitrous oxide fluxes correlated negatively with *nosZ* gene abundance (*r* = -0.59, *P* < 0.05) and soil pH (*r* = 0.65, *P* < 0.01), and positively with soil temperature (*r* = 0.57, *P* < 0.05) in OA. In the UC area, N_2_O fluxes correlated positively with total enzyme activity (*r* = 0.60, *P* < 0.05), with glucuronidase activity (*r* = 0.58, *P* < 0.05), with glucosaminidase activity (*r* = 0.67, *P* < 0.01), and with phosphatase activity (*r* = 0.56, *P* < 0.05). In the OA area N_2_O fluxes correlated positively with phosphatase activity (*r* = 0.58, *P* < 0.05). N_2_O fluxes in the UC area shown as CO_2_-equivalents was higher than that in OA area (**Table 2**).

## Discussion

The cork oak trees had a significant effect on soil properties and subsequent soil EEAs, on the abundance of microbes, and finally on the non-CO_2_ net GHG fluxes. In this study soil CH_4_ uptake was generally activated in autumn when soil moisture was higher and temperature lower than in summer. Trees are known to affect soil CH_4_ consumption, but whether this is due to tree effects on microbial CH_4_ oxidation or soil gas diffusivity is not known (Menyailo, 2007; Menyailo et al., 2010). Oak canopy increased soil moisture, which could explain the stronger negative correlation found between methane fluxes and *pmoA* gene abundance in the UC area compared to the OA area. It is possible, that the dryness in the OA area limited the activity and growth of methanotrophs. Thus, even at the highest water content, moisture did not limit the activity of methanotrophs indicating good availability of oxygen and methane. SWC and associated gas diffusivity are known to affect abundance and activity of methanotrophs (Borjesson et al., 2004; Einola et al., 2007). However, there is evidence for the presence of anaerobic microsites in the studied soils because net CH_4_ emissions were also observed, showing that in some moisture and temperature conditions CH_4_ production (activity of methanogens) exceeded CH_4_ oxidation (activity of methanotrophs). The net release of CH_4_ correlated positively with temperature and soil organic matter and carbon indicating that these factors favored methanogens over methanotrophs. However, Type Ia methanotrophs especially in the UC areas had a significant role in reducing of CH_4_ emissions and in the consumption of atmospheric CH_4_ since their abundance was affected by seasonal variation and correlated with CH_4_ eﬄux. Input of organic carbon by trees in UC area increased CH_4_ cycling, and therefore a positive correlation in UC area but not in OA area can be explained. An increase in organic matter supports the activity of heterotrophic microbes as seen here by the higher enzyme activities in the UC area as compared to the OA area. It is likely that the availability of low molecular weight organic substrates needed for methanogenesis was higher in the UC area resulting from the higher enzyme activities found there. An increase in soil temperature further supported net CH_4_ release in the present study. This is associated with higher microbial decomposition processes and oxygen consumption at higher temperatures, which can create anaerobic microsites in the clay-rich soil. It is noteworthy that CH_4_ fluxes in the UC and OA areas did not differ much. We would expect higher CH_4_ production in UC area rather than in OA area. Evidently the higher CH_4_ oxidation in the UC area discussed above counteracted the possible higher CH_4_ production there.

Methanotrophs in the study site belonged to Type Ia methanotrophs. Type I methanotrophs are usually found in extreme conditions where competition survival strategy supports their fast response to improved substrate availability (Ho et al., 2013). Moreover, Type I methanotrophs grow in a wide temperature range, from thermophilic (Bodrossy et al., 1997; Tsubota et al., 2005) to psychrophilic (Liebner et al., 2009; Graef et al., 2011) conditions. In this site, soil temperature varied substantially from 12.7 to 27.9°C, which could favor the occurrence of Type I methanotrophs over the other types. In addition to the temperature related selection, potential internal methane source in the soil as reflected as CH_4_ emissions, might have selected for presumably low affinity Type I methanotrophs in this site. However, the PCR assay used for USC(α) methanotrophs (Kolb et al., 2003) might not have recognized all high-affinity atmospheric CH_4_ oxidizers living in this site. These methanotrophs could have been detected more recently generated primer set with broader specifity for USC(α) (Degelmann et al., 2010).

Nitrous oxide uptake from the atmosphere has been explored in few reports even under dry conditions when gas diffusivity is good (Rosenkranz et al., 2006; Goldberg and Gebauer, 2009). In theory, the dry conditions when oxygen availability is high should not support nitrous oxide reduction (Morley et al., 2008). Rosenkranz et al. (2006) linked negative fluxes in Mediterranean forest soil to very low N availability and high soil C content, and considered aerobic denitrification by heterotrophic denitrifiers as a possible pathway for N_2_O uptake. In our soil, higher soil moisture, higher *nosZ* gene abundance, higher total enzyme activities, and higher N_2_O fluxes (emissions) were concurrent within UC area. Mineralization of SOM and exudates from tree roots in the UC area produced more soluble carbon to fuel denitrification. However, nitrate content was similar in both areas. We have no data on nitrification activity and nitrate uptake by plants, which hampers a concise conclusion about the nitrate turnover and availability in soils. In the OA area there was a positive correlation between N_2_O fluxes and *nosZ* gene abundance in contrast to the UC area. The primer set used for enumeration of *nosZ* genes did not cover *nosZ*-II genes. However, the typical N_2_O consuming *nosZ* genes detected in our study had a significant role in N_2_O consumption, since their abundance was correlated with N_2_O flux and affected by seasonal variation. We observed a positive correlation between SOM input in the UC area and catalase activity of four studied enzymes that degrade SOM and provide energy (C) and nutrients (N and P) for ecosystem functioning. These catalases also correlated positively with N_2_O flux in UC area. Since denitrifiers require organic carbon for growth, a correlative link between N_2_O flux and enzyme activities can be explained by their heterotrophic lifestyle.

Moreover, in this study N_2_O uptake was correlated with lower EEA, lower C and N supply and lower soil moisture. Positive correlations between N_2_O fluxes and soil enzyme activities, especially in UC area, could be explained by higher SOM input into UC area. However, in the UC area with higher water content and substrate availability for denitrification, more of the produced N_2_O could be reduced to N_2_ and therefore gene abundance of *nosZ* did not reflect the overall denitrification. The EEAs are not connected directly to metabolism of nitrous oxide or bacterial denitrification. However, EEAs may provide a clue about the soil microbial activity in general, which is correlated with nitrous oxide fluxes. These correlations need to be evaluated critically since these linkages may be simply co-incidental without a real metabolic connection to each other. The impact of trees on soil properties (SWC, SOM, litter fall, root density) and a strong positive correlation between SOM and both CH_4_ and N_2_O eﬄuxes were previously reported (Shvaleva et al., 2014). The current study was able to link the abundance of methanotrophs with CH_4_ fluxes in UC area, and the abundance of N_2_O consuming bacteria in OA area.

Nitrous oxide uptake was detected in 60% of all studied time-points. While measuring such small fluxes close to the detection limit of the gas chromatograph used, it is important to evaluate if the equipment is sensitive enough to detect N_2_O uptake. The GC and detectors used were accurate enough to measure such small N_2_O fluxes. Most of measured N_2_O fluxes were above minimum detectable flux. However, the measurements performed for non-CO_2_ GHG fluxes didn’t cover whole ecosystem GHG fluxes including processes in the phyllosphere. The tree stand itself contributes to the GHG balance by CO_2_ sequestration through photosynthesis. In addition, trees are a transpiration channel from soil to atmosphere and it has been shown that plants are capable of CH_4_ emissions (Keppler et al., 2006; Carmichael et al., 2014) and in some circumstances CH_4_ uptake is possible by plants (Sundqvist et al., 2012). Moreover, N_2_O emissions from plants were reported recently, with a rate comparable with soil N_2_O emissions, by ammonia oxidizing bacteria on leaf surfaces (Bowatte et al., 2015). Therefore our measured soil-related non-CO_2_ GHG balances between UC and OA areas might be underestimated, and we can’t be completely certain of the total balance of all GHG produced and consumed in these sites. Similar non-CO_2_ GHG balances were also earlier examined in this same study-site (Shvaleva et al., 2014). However, earlier in another montado site higher CH_4_ uptake compensated N_2_O emission, which kept non-CO_2_ balance negative (Shvaleva et al., 2011). This emphasizes spatial and seasonal variation of GHG eﬄuxes in montado ecosystems. However, if future climatic conditions support tree decline, soil related nitrous oxide emissions might be reduced from Mediterranean montado ecosystems, provided that understory vegetation and soil conditions remain similar to OA area.

## Conclusion

Oak tree cover had an effect on soil properties, soil enzymatic activities, and the abundance of CH_4_ and N_2_O metabolizing bacteria and as a consequence, on CH_4_ and N_2_O fluxes. Correlation between soil-atmosphere CH_4_ exchange and abundance of Type I *pmoA* genes under tree canopies, and correlation between N_2_O exchange and abundance of *nosZ* genes in OAs suggests that these microbial groups may contribute to most of the gasses consumed in evergreen oak woodlands. Oak trees exert these effects on a functional group of soil micro-organisms through the complex interactions between plants, microorganisms, and soil characteristics (SWC, SOM, root density, litter fall, and enzyme activities). Our results suggest that oak tree vegetation does not change mean soil CH_4_ uptake, but significantly increases mean N_2_O fluxes and this neutralizes the soil non-CO_2_ uptake in Mediterranean oak forests, and it can even turn the soil non-CO_2_ GHG balance from negative to positive when compared to non-oak tree vegetated surfaces.

## Conflict of Interest Statement

The authors declare that the research was conducted in the absence of any commercial or financial relationships that could be construed as a potential conflict of interest.
