# Supplementary material for: Environmental and microbial factors influencing methane and nitrous oxide fluxes in Mediterranean cork oak woodlands: trees make a difference
Source: Front Microbiol. 2015 Oct 14;6:1104. doi: 10.3389/fmicb.2015.01104 (PMC4604323; doi:10.3389/fmicb.2015.01104)
Supplement: Supplementary file 1 [file Table_1.DOCX]

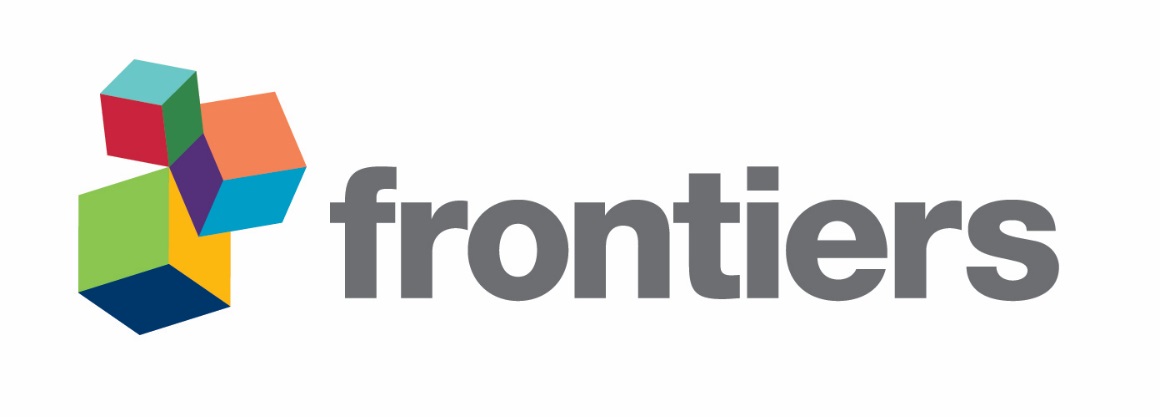


Supporting information for,

**Microbial factors controlling methane and nitrous oxide fluxes in Mediterranean cork oak woodlands: trees make a difference**

Alla Shvaleva^1^*^¥^*, Henri Siljanen^2^*^¥^*, Alexandra Correia^3^, Filipe Costa e Silva^3^, Richard Lamprecht^2^, Joaquim Miguel Costa^1^, Raquel Lobo-do-Vale^3^, Catarina Bicho^1^, David Fangueiro^3^, Margaret Anderson^4^, João Santos Pereira^3^, Ute Skiba^4^, Maria Manuela Chaves^1^, Cristina Cruz^5^, Pertti J, Martikainen^2^

**Correspondence:** Corresponding author: henri.siljanen@uef.fi

**TABLE S1.** Precipitation (mm) and air temperature (°C) measured during the study period, i.e. in May, August, October, November and December, 2011.

| Meteorological data | May  (end of rain period) | August  (dry extreme conditions) | October  (first autumn rain events) | November  (wet extreme conditions) | December (stabilize wet conditions) |
| --- | --- | --- | --- | --- | --- |
| Precipitation (mm) | 111 | 8 | 92 | 149 | 23 |
| Air temperature, ºC | 19 | 21 | 20 | 12 | 10 |

**TABLE S2**. Soil extracellular enzymes assayed in this study and their functions.

| Enzyme | Abbrevation | EC number | Function |
| --- | --- | --- | --- |
| Acid phosphatase | Pho | 3.1.3.2. | Release of phosphate from ester-bonded P |
| N-acetyl-ß-D-glucosaminidase | Nag | 3.2.1.14 | Release of N-acetyl- ß-D-glucosaminide from chiton oligomers |
| ß-glucuronidase | Glr | 3.2.1.31 | Release of glucuronic acid |
| ß-glucosidase | Gls | 3.2.1.21 | Release of glucose from glucosides, cellobiose |
| 1,4-ß-Cellobiosidase | Cel | 3.2.1.91 | Release of cellobiose from non-reducing end of cellulose chains |
| 1,4- ß-Xylosidase | Xyl | 3.2.1.37 | Release of xylose from short xylan oligomers |
| Laccase | Lac | 1.10.3.2 | Phenole compounds oxidation and molecular oxygen reduction to water |

**Table S3.** Primers and annealing temperatures used in this study.

| Primer | Target | Sequence 5' - 3' | Tann | Reference |
| --- | --- | --- | --- | --- |
| A189f | Type I/II methanotrophs, RA14, Upland soil cluster a, *pmoA* | GGNGACTGGGACTTCTGG | 62-52 | Holmes et al., 1995 |
| A682 * | Type I/II methanotrophs, *pmoA* | GAASGCNGAGAAGAASGC | 62-52 | Holmes et al., 1995 |
| mb661 ** | Type I/II methanotrophs, RA14, Upland soil cluster a, *pmoA* | CCGGMGCAACGTCYTTACC | 62-52 | Costello & Lidstrom, 1999 |
| Mb601r * | Type Ia *pmoA* | ACRTAGTGGTAACCTTGYAA | 54.0 | Kolb et al., 2003 |
| Mc468r * | Type Ib *pmoA* | GCSGTGAACAGGTAGCTGCC | 64 | Kolb et al., 2003 |
| II223f | Type II *pmoA* | CGTCGTATCTGGCCGAC | 69,5 | Kolb et al., 2003 |
| II646r | Type II *pmoA* | CGTGCCGCGCTCGACCATGYG | 69,5 | Kolb et al., 2003 |
| Forest675r * | Forest clones, Upland soil cluster a, *pmoA* | CCYACSACATCCTTACCGAA | 67 | Kolb et al., 2003 |
| mmoXLF | *Methylocella* *mmoX* (sMMO) | GAAGATTGGGGCGGCATCTG | 68 | Rahman et al., 2011 |
| mmoXLR | *Methylocella* *mmoX* (sMMO) | CCCAATCATCGCTGAAGGAGT | 68 | Rahman et al., 2011 |
| nosZ2F | *nosZ* | CGCRACGGCAASAAGGTSMSSGT | 60 | Henry et al. 2006 |
| nosZ2R | *nosZ* | CAKRTGCAKSGCRTGGCAGAA | 60 | Henry et al. 2006 |

* primer used with A189f primer

** primer used with A189f after semi-nested amplification with A189f and A682r primers
